# Family structure and risk factors for schizophrenia: case-sibling study

**DOI:** 10.1186/1471-244X-4-41

**Published:** 2004-11-27

**Authors:** Jari K Haukka, Jaana Suvisaari, Jouko Lönnqvist

**Affiliations:** 1 Department of Mental Health and Alcohol Research, KTL, National Public Health Institute, Mannerheimintie 160, FIN-00300 Helsinki, Finland

## Abstract

**Background:**

Several family structure-related factors, such as birth order, family size, parental age, and age differences to siblings, have been suggested as risk factors for schizophrenia. We examined how family-structure-related variables modified the risk of schizophrenia in Finnish families with at least one child with schizophrenia born from 1950 to 1976.

**Methods:**

We used case-sibling design, a variant of the matched case-control design in the analysis. Patients hospitalized for schizophrenia between 1969 and 1996 were identified from the Finnish Hospital Discharge Register, and their families from the Population Register Center. Only families with at least two children (7914 sibships and 21059 individuals) were included in the analysis. Conditional logistic regression with sex, birth cohort, maternal schizophrenia status, and several family-related variables as explanatory variables was used in the case-sibling design. The effect of variables with the same value in each sibship was analyzed using ordinary logistic regression.

**Results:**

Having a sibling who was less than five years older (OR 1.46, 95% CI 1.29–1.66), or being the firstborn (first born vs. second born 1.62, 1.87–1.4) predicted an elevated risk, but having siblings who were more than ten years older predicted a lower risk (0.66, 0.56–0.79).

**Conclusions:**

Several family-structure-related variables were identified as risk factors for schizophrenia. The underlying causative mechanisms are likely to be variable.

## Background

Several factors relating to family structure have been suggested as risk factors for schizophrenia. They include sibship size, number of older siblings, birth order in the sibship, age difference to older siblings, and age-at-onset, and sex of the affected siblings. These family-related risk factors may provide useful hints about the nature of environmental and genetic risk factors for schizophrenia [1].

In several studies, siblings of female probands have had a higher risk of developing schizophrenia than siblings of male probands in several studies, with the number of cases varying from 123 to 500 [2-7]. The effect of the age-at-onset of proband is less clear. Some studies have detected no effect [5,8], but in one, early age-at-onset of proband predicted a higher risk for siblings [4].

There are mixed results about the effect of birth order on risk. Two studies reported lowest risk for the firstborns [9,10], and in a Swedish population based study with 167 cases, male subjects fourth or higher in birth order had over a three-fold risk compared to males with lower birth order [11]. On the other hand, a very large scale Danish cohort study involving 2669 cases showed no effect for birth order [12]. A significantly higher risk for first-born males was reported from a Finnish cohort study with 100 cases [13], and in accordance with this, a recent study from Pakistan with 453 cases reported highly significant excess of firstborns among patients with schizophrenia [14].

The number and age of older siblings at the time of birth is hypothesized to be connected to the risk of schizophrenia through infection pressure. In a Swedish register-based study (270 cases), having siblings who were 3–4 years older was associated with increased risk (relative was 2.02) [10]. The Danish cohort showed no effect of birth order on risk, but found that having less than two years age difference to the nearest older or younger sibling increased the risk of schizophrenia [12]. However, in an Irish case-control study with 2969 cases and 5904 controls, the number of older siblings did not predict a diagnosis of schizophrenia over neurosis [15]. Several recent studies have found that advanced paternal age is a risk factor for schizophrenia [16-18].

All risk factors concerning birth order, family size, number of older or younger siblings, and paternal and maternal age are correlated. For example, the number of older siblings is dependent on both the individual's birth order and family size, which are connected to maternal age. If all these correlated variables are not in the model simultaneously, spurious associations may be found. Therefore, a large, population-based data set that contains a sufficient number of sibships with variable structures is needed to analyze family-related variables. Our aim was to investigate which of these variables were risk factors for schizophrenia in Finland among those who were born between 1950 and 1976.

## Methods

### Register data, study design and statistical analyses

Using the Finnish Hospital Discharge Register of Finland, we obtained information on all hospitalizations with a diagnosis of schizophrenia (ICD-8 295*, ICD-9 295*, ICD-10 F20*). The information was sought for all Finns born between 1950 and 1976 for the period between 1969 and 1996. Sibship was defined as a group of individuals having the same mother. Since many of the variables we examined are related to maternal characteristics, we considered it essential that each sibship has the same mother. It should be noticed that generally the mother has a stronger influence on the child than the father especially in early childhood: e.g. mother's womb provides the fetal environment, and after birth the mother has more of a close physical relationship to the child through breast feeding. Siblings and parents of the probands were identified using the data from the Population Register Centre, and their information was then linked back to the Hospital Discharge Register to obtain data on relatives' hospitalizations. Only those who were born between 1950 and 1976 were qualified as probands, but their younger siblings were included in analyses as family members.

We assumed our ascertainment was complete, because the two registers used, the Population Register and the Finnish Hospital Discharge Register, are nationwide and accurate [19]. The reliability of register diagnoses of schizophrenia is good [20-22] and according to previous studies, more than 95 percent of individuals with schizophrenia receive hospital treatment at some point in their lives [22].

Altogether, we identified 17549 sibships with 35481 siblings, of whom 31004 were born between 1950 and 1976. There were 12 siblings in the largest family, and the highest number of cases in one family was six (Table 3). All families that contained only one child (n = 7947) were excluded from the study because almost all variables we investigated required there to be more than one child in the family.

**Table 3:** A family study of schizophrenia in Finland in birth cohorts born from 1950 to 1976. Family structure of families (N = 2605) with at least one sibling with schizophrenia; also siblings born before 1950 and after 1976 are included.

N. of cases in the family	Family size										
	2	3	4	5	6	7	8	9	10	11	12
1	3289	2012	984	369	171	62	22	9	5	1	0
2	282	271	190	82	35	16	12	5	1	0	0
3	0	22	26	11	10	8	4	0	1	0	1
4	0	0	4	2	1	1	1	0	0	0	0
5	0	0	0	1	0	1	0	0	0	0	0
6	0	0	0	0	0	0	1	1	0	0	0

We used two types of explanatory variables: variables that were unique for each person, and variables that had the same value for all members of the sibship. The unique variables were sex, birth cohort, birth order, number and ages of older siblings at the time of birth, and the age of mother and father at the time of birth, and the shared variables were the lowest age-at-onset in sibship, sex of the proband with lowest age-at-onset in the sibship, and the sibship size, and whether the mother had schizophrenia or not.

### Study design and statistical analyses

We investigated the effect of unique variables using the case-sibling design [23], in which each case was matched with one or more unaffected siblings. We modeled the data with conditional logistic regression using each case in the family as the conditioning unit, which is the standard method for case-sibling design [23].

The sibling with the lowest age-at-onset of schizophrenia was the first case or proband and all unaffected siblings born between 1950 and 1976 and alive at age when first sibling had his/her first illness episode, were chosen as controls. Thus, the matching was carried out with respect to age-at-onset. If there were later cases in the family, controls were chosen in the same way but excluding those siblings who already had developed schizophrenia. Thus, a sibling could serve as a control before developing schizophrenia. The number of cases with at least one control was 9013 and the number of controls was 12046. As shown in Table 4, our data included a substantial number of families with more than 5 siblings.

**Table 4:** Basic characteristics of sibships.

		no schizophrenia (%)		schizophrenia (%)		All
**Sex**	male	4636	47	5207	52	9843
	female	7410	66	3806	34	11216
**Siblings under 5 years**	no	5496	52	4876	48	10372
	yes	6550	61	4137	39	10687
**Siblings 5–10 years**	no	7727	52	7172	48	14899
	yes	4319	70	1841	30	6160
**Siblings over 10 years**	no	10094	55	8377	45	18471
	yes	1952	75	636	25	2588
**Birth order**	1	2934	44	3781	56	6715
	2	4297	57	3260	43	7557
	3	2629	68	1262	32	3891
	4+	2186	75	710	25	2896
**Birth cohort**	1950–1955	3756	51	3648	49	7404
	1956–1960	3566	57	2689	43	6255
	1961–1965	2825	62	1748	38	4573
	1966–1976	1899	67	928	33	2827
**Maternal age**	under 20	441	45	546	55	987
	20–25	2509	50	2462	50	4971
	26–30	3419	56	2692	44	6111
	31–35	2896	62	1778	38	4674
	over 35	2781	64	1535	36	4316
**Paternal age**	under 20	101	49	106	51	207
	20–25	1281	49	1355	51	2636
	26–30	2727	53	2413	47	5140
	31–35	2765	60	1879	40	4644
	over 35	4159	63	2407	37	6566
	not available	1013	54	853	46	1866
**Sex of youngest proband**	male	6880	57	5187	43	12067
	female	5166	57	3826	43	8992
**Lowest age-at-onset in sibship**	under 20	2797	54	2351	46	5148
	20–25	4410	57	3350	43	7760
	26–30	2930	59	2014	41	4944
	over 30	1909	60	1298	40	3207
**Family size**	2	3623	46	4233	54	7856
	3	3623	59	2551	41	6174
	4	2373	65	1284	35	3657
	5+	2427	72	945	28	3372
**Mother schizophrenic**	no	11537	57	8561	43	20098
	yes	509	53	452	47	961

Shared variables, which were the lowest age-at-onset in sibship, sex of the proband with the lowest age-at-onset in the sibship, and the sibship size, were modeled using ordinary logistic regression without conditioning. The probands, who were the affected siblings with the lowest age-at-onset in the families, were not included in this model.

We tested the significance of the interactions between sex and other variables, and interactions between sex of the proband and other variables using likelihood ratio tests.

## Results

In the conditional logistic regression, females had lower risk of developing schizophrenia (Table 1). Having siblings who were less than five years older was associated with increased risk of schizophrenia, while having siblings who were more than ten years older was associated with a lower risk of schizophrenia. The firstborn had a higher risk of schizophrenia than later-borns. Young maternal age was associated with increased risk of schizophrenia, while young paternal age decreased risk.

**Table 1:** The effect of unique family-related variables on the odds of developing schizophrenia: results from the multivariate conditional logistic regression model which used the matched case-sibling design, odds ratios with 95% confidence intervals.

**Sex (female vs. male)**	0.507(0.478, 0.537)
**Siblings who were less than 5 years older (yes vs. no)**	1.463(1.288, 1.661)
**Siblings who were 5–10 years older (yes vs. no)**	0.905(0.800, 1.025)
**Over 10 years older siblings (yes vs. no)**	0.661(0.556, 0.785)
**Birth order**	
1	(reference)
2	0.618(0.535, 0.715)
3	0.542(0.441, 0.666)
4+	0.504(0.382, 0.667)
**Birth cohort**	
1950–1955	(reference)
1956–1960	1.143(1.032, 1.267)
1961–1965	1.199(1.015, 1.415)
1966–1976	0.956(0.746, 1.224)
**Maternal age**	
under 20	(reference)
20–25	0.794(0.671, 0.939)
26–30	0.688(0.555, 0.852)
31–35	0.614(0.470, 0.803)
over 35	0.605(0.433, 0.845)
**Paternal age**	
under 20	(reference)
20–25	1.382(0.990, 1.927)
26–30	1.432(1.004, 2.043)
31–35	1.340(0.915, 1.963)
over 35	1.332(0.880, 2.015)
not available	0.213(0.018, 2.491)

In the ordinary logistic regression, siblings had higher risk if the proband had early, under 20 years, age at onset (Table 2). A sibship size of four or more was associated with increased risk of schizophrenia, as was having a mother with schizophrenia. The effect of parental age and having siblings who were less than 5 years older in the sibship disappeared in the ordinary logistic regression, but the effect of being the first born and having siblings who were more than 10 years older remained the same.

**Table 2:** The effect of shared family-related variables on siblings' odds of developing schizophrenia: results from the multivariate logistic regression where the proband was removed from the analysis, odds ratios with 95% confidence intervals. Unique family-related variables (sex, ages of other siblings, birth order, birth cohort, maternal age, and paternal age) were also included in models as background variables.

**Sex of the youngest proband (female vs. male)**	0.983(0.864, 1.117)
**Lowest age-at-onset in the sibship**	
under 20	(reference)
20–25	0.574(0.497, 0.664)
26–30	0.323(0.267, 0.391)
over 30	0.156(0.116, 0.209)
**Family size**	
2	(reference)
3	1.128(0.943, 1.349)
4	1.282(1.043, 1.575)
5+	1.286(1.019, 1.623)
**Mother schizophrenic(no vs. yes)**	1.822(1.431, 2.321)

None of the tested interactions were significant.

We investigated the effect of proband's sex and age-at-onset, birth order, age difference to siblings, birth cohort, and parental ages on the risk of developing schizophrenia. Proband's early age-at-onset and female sex increased the risk of schizophrenia among siblings. Male sex, having siblings who were less than 5 years older, and being the firstborn also increased the risk of schizophrenia, while having over 10 years older siblings and small family size decreased it.

The higher risk of schizophrenia among the firstborns confirms the findings from some other recent studies. The most likely explanation is the increased risk of pregnancy and delivery complications among primiparous (first delivery) women. In the 1966 and 1986 Northern Finland birth cohorts, primiparity was a risk factor for having a low birth weight infant and for perinatal mortality [24]. In an earlier Finnish cohort study, infants of multiparous women were significantly heavier than infants of primiparous women [25]. As low birth weight and some obstetric complications increase the risk of later development for schizophrenia [26], they may explain the increased risk of schizophrenia among the firstborns. Having siblings who were less than five years older was a risk factor for schizophrenia in our study. Sham et al. found that having siblings who were 3–4 years older was a risk factor for schizophrenia [10], while Westergaard et al. found that a short interval to the nearest older sibling was a risk factor for schizophrenia [12]. Both suggested that common infections brought to the family by young siblings, causing fetal or early childhood infections, could lie behind the association [10,27]. Consistently with this, Brown et al found in a cohort study that second trimester exposure to respiratory infections increased the risk of later development of schizophrenia spectrum disorders two-fold [28].

## Discussion

Our results concerning the effect of paternal and maternal age are not comparable to previous studies [16-18], because all patients who did not have siblings were excluded from the analysis. Our findings suggest that in families with at least two children, young maternal age at birth is a risk factor for schizophrenia. This could be linked to obstetric complications. However, although some studies have found that obstetric complications are more common among adolescent mothers [29], this is not the case in Finland [24,30]. Thus, obstetric complications probably do not explain why the offspring of young mothers have an increased risk of schizophrenia.

Consistently with a recent meta-analysis [31], the risk of schizophrenia was 50 percent lower for females than males. If risk factors for schizophrenia are equally distributed among females and males but females are for some reason more resilient to them, it could be assumed that females who develop schizophrenia have more schizophrenia-predisposing genes than males. This would explain why siblings' risk of schizophrenia was higher when the proband was female.

Our results showed that the higher was the age-at-onset of the first proband in the family, the lower was the siblings' risk (Tables 1 and 2), which is in line with previous family studies [5,32,33] and also with studies that found an earlier age-at-onset among patients with familiar rather than sporadic schizophrenia [34]. Also, a study of parents of patients with childhood- and adult-onset schizophrenia found that parents of patients with childhood-onset schizophrenia had a significantly higher morbid risk of schizophrenia spectrum disorders than parents with adult-onset schizophrenia [35]. All these findings are consistent with the hypothesis that in schizophrenia, as in many other multifactorial neuropsychiatric diseases [36], early age at onset is a marker of greater genetic vulnerability to the disorder.

A case-sibling design has some limitations. It can only provide approximate estimates of relative risk. In our analyses only families with two or more siblings were informative, and therefore our results are not applicable to one child families. However, whether matching was taken into account or not did not largely affect the results.

Our study was based on a nationwide, representative sample of patients with schizophrenia and their siblings, and our sample size was considerably larger than in any of the previous studies. Register-based information is not as accurate as information based on face-to-face interview, but Finnish registers are quite reliable. The two registers we used, the National Population Register and the National Hospital Discharge Register, are nationwide. The National Hospital Discharge Register covers all public and private hospitals in Finland, and the National Population Register registers all Finnish citizens. However, as information on parents enabling the identification of sibships first appeared in the Population Register only in 1974, sibship data may be more accurate in the younger cohorts. Information in both registers is accurate. Some proportion of siblings of the study population are really half-siblings, but even in this case, the case-sibling design is applicable, because it does not assume that familial aggregation is due to a genetic mechanism. When entries in the National Hospital Discharge Register were compared with data on hospital case notes, the primary diagnosis in the register and hospital case notes was identical in 99 percent of cases for schizophrenia and in 98 percent of cases for all mental disorders [19]. The reliability of register diagnoses of schizophrenia is also good, with a greater propensity for false-negatives than false-positives [20-22,37].

## Conclusions

We investigated concurrently several family-related factors which independently have been suggested to be risk factors for schizophrenia. We were able to verify some of the previous associations, while others were refuted. The risk factors we identified probably have different underlying causative mechanisms. For example, the risk associated with being the firstborn might be explained by increased risk to obstetric complications, while the combination of decreased risk among females but increased risk among siblings of female probands might be explained by genetic factors. The risk factors we identified should be studied further in samples that include more detailed information on possible causative factors, such as infections and obstetric complications.

## Competing interests

The author(s) declare that they have no competing interests.

## Authors contributions

JH participated in the design of the study, performed the data-analysis and drafted the manuscript. JS participated in the design of the study, and the drafting of the manuscript. JL participated in the design of the study and its coordination. All authors read and approved the final manuscript.

## Pre-publication history

The pre-publication history for this paper can be accessed here:


